# The Significance of the Alter miR let-7a and miR-335 Expression Level Regulating the CCR7/CCL19 Axis as Potential Biomarkers of Tumor Progression in NSCLC

**DOI:** 10.3390/jcm11030655

**Published:** 2022-01-27

**Authors:** Kamila Baran, Jacek Kordiak, Sławomir Jabłoński, Adam Antczak, Ewa Brzeziańska-Lasota

**Affiliations:** 1 Department of Biomedicine and Genetics, Medical University of Lodz, 92-213 Lodz, Poland; ewa.brzezianska@umed.lodz.pl; 2 Department of Thoracic, General and Oncological Surgery, Medical University of Lodz, 90-549 Lodz, Poland; jacek.kordiak@umed.lodz.pl (J.K.); slawomir.jablonski@umed.lodz.pl (S.J.); 3 Department of General and Oncological Pulmonology, Medical University of Lodz, 90-153 Lodz, Poland; adam.antczak@umed.lodz.pl

**Keywords:** non-small lung cancer, real-time polymerase chain reaction, *CCR7*, *CCL19*, miR-335, miR let-7a

## Abstract

The chemokine receptor 7/C-C ligand 19 chemokine (CCR7/CCL19) has been implicated in the development and progression of NSCLC. Its expression is regulated by various epigenetic factors including miRNAs. The aim of this study was to assess the expression of *CCR7/CCL19* in cancer tissue in relation to that of miRNAs (miR-let-7a, miR-335) as transcriptional regulators. The expression of the tested miRNAs was also evaluated in serum exosomes. Sixty patients (*n* = 60) were enrolled in the study. The total expression of the studied mRNA and miRNAs were evaluated using qPCR. Tumor tissue fragments, macroscopically unchanged adjacent tissue, and serum were used as controls. Higher *CCR7* and *CCL19* mRNA expression levels were observed in tumor tissue compared to control. According to stages of the disease (AJCC tumor staging), the greatest expression level of the studied genes’ mRNA was observed in patients with stage III. In NSCLC patients, lower miR let-7a expression level was observed in tumor tissue compared to serum; however, miR-335 expression level was higher (*p* < 0.05). The expression level of miR-335 positively correlated with tumor size (T features according to pTNM staging) and AJCC tumor staging, while miR let-7a had a negative correlation (*p* > 0.05) with liquid biopsy. Significantly greater miR-335 expression level and lower miR let-7a expression level in serum were observed in patients with metastases to lymph nodes. Our findings reveal a significant correlation between the expression levels of the mRNA of the studied genes and miRNAs. Changes in miR-335 and miR let-7a expression levels in the serum exosomes of NSCLC patients in relation to lymph node metastases and tumor stage may serve as a non-invasive molecular biomarker of tumor progression.

## 1. Introduction

Lung cancer is one of the most commonly diagnosed cancers and the leading cause of cancer-related mortality. According to WHO data, 2,206,771 new cases of lung cancer were recorded in 2020, which accounts for 11.4% of all cancer cases [1]. It was as responsible for the deaths of approximately 1,796,144 people worldwide last year, 27,444 of which were from Poland. The most frequently diagnosed histological type is non-small cell lung cancer (NSCLC), which accounts for 85% of cases. The high mortality of lung cancer, including NSCLC, is caused mainly by the late diagnosis of patients with advanced-stage cancer. A better understanding of the molecular mechanisms responsible for the development and progression of NSCLC will reveal biomarkers that can be used for diagnosis. 

The system of chemokines and their receptors plays an important role in the progression of cancer. The discovery of the CC chemokine receptor 7 (CCR7) in the membrane of lung cancer cells suggests that it may be utilized to migrate along the gradient of C-C motif chemokine 19 (CCL19) and chemokine 21 (CCL21) towards the lymph node and subsequently cause lymph node metastasis [2]. Furthermore, CCR7 and its ligands have been implicated in the occurrence and progression of NSCLC [3,4]. It was revealed that CCR7 expression is induced by hypoxia, which enhances the migration and invasion of lung cancer cells. Furthermore, CCR7 play a key role in the regulation of cell proliferation, apoptosis, and epithelial-mesenchymal transition (EMT) via the ERK and NF-κB pathway [3,5,6]. The recent study revealed that matrine, alkaloid isolated from plants of *Saphora* species, induces cell apoptosis in NSCLC cell line via targeting CCR7 [7]. Moreover, inhibition of CCR7 expression by metrine enhances effects of anticancer treatment, such as cisplatin, 5-fluorouracil, and paclitaxel in vitro. Thus, CCR7 may serve as a novel therapeutic target for NSCLC. Furthermore, the study performed in murine models of lung cancer demonstrated that local and systemic administration of CCL19 combined with IL-7 is beneficial as potent anti-cancer strategy by stimulation of the immune response [8]. Other research revealed that tumor microenvironment cells, such as fibroblastic stromal cells, produce CCL19, which promote local antitumor T-cell responses and restrain the growth of lung carcinoma [9]. Moreover, Itakura et al. [10] revealed that a high *CCL19* expression level was a good prognostic factor in lung adenocarcinoma. Hence, the diagnostic value of the *CCR7/CCL19* axis in lung tumorigenesis remains controversial [2,10].

It has been revealed that microRNAs (miRNAs) may regulate the expression of the messenger ribonucleic acid (mRNA) of chemokines or their receptors and play a role in various cellular processes implicated in the development and progression of NSCLC [11,12]. miRNA are single-stranded RNA chains of approximately 22 nucleotides that do not encode proteins. These molecules negatively regulate the post-transcriptional level of target mRNAs by binding to the 3′-untranslated region (UTR), resulting in the degradation of mRNA or translation inhibition [13]. They can exert oncogenic and tumor-suppressive effects on cancer cells influencing tumor microenvironment [14]. Moreover, oncogenic miRNAs may participate in tumor progression by their transfer of exosomes to other regions of the body, where the promotion of metastasis occurs. Dysregulation in miRNAs expression during tumorigenesis may be caused by various mechanisms, including the amplification or deletion of miRNA genes, abnormal transcriptional control of miRNAs, and dysregulated epigenetic changes [15]. An alternation in miRNA expression levels may serve as diagnostic and/or prognostic biomarkers in NSCLC in liquid biopsy. 

This research is a continuation and extension of our earlier study, the aim of which was to evaluate changes in the expression level of *CCR7* and *CCL19* in tumor tissue and of two regulatory miRNAs of these genes (miR let-7a and miR-335) in serum exosomes [16]. A particularly valuable element of this study is the measurement of miRNA expression level in tumor tissue. It also compares the expression profiles of selected miRNAs originating from the exosomes and tumor tissue of NSCLC patients and analyzes the differences in the expression of the chosen miRNAs, *CCR7* and *CCL19,* with regard to selected clinical features of NSCLC patients.

## 2. Experimental Section

### 2.1. Subjects

The study cohort consisted of 60 patients with diagnosed NSCLC, 25 women and 35 men, aged 51 to 81 (mean age 67.13 ± 7.14 years). Lung resection (pulmonectomy or lobectomy) in patients was performed at the Department of Thoracic Surgery, General and Oncological Surgery, Military Medical Academy Memorial Teaching Hospital of the Medical University of Lodz–Central Veterans’ Hospital, Lodz, Poland during the years 2018–2019. The clinical features of the patients with diagnosed NSCLC are summarized in Table 1. 

### 2.2. Tissue and Serum Collection

Primary tumor tissue fragments from diagnosed NSCLC patients were obtained from the middle of the lesion (approximately 100 mg). Adjacent macroscopically unchanged tissue (approximately 100 mg taken at a distance of 2 cm from the primary focus) was used as the control. The resected primary tumors were classified as either squamous cell carcinoma (SCC) or adenocarcinoma (AC). The tissue samples were subjected to a postoperative histopathological evaluation and classified according to the AJCC and TNM staging systems (pTNM) [18]. The histopathological characteristics of the biological materials is shown in Table 1.

Serum from all (*n* = 60) study patients with diagnosed NSCLC were collected before the surgery. Sera from 45 patients (potentially healthy volunteers) were used as controls.

The biological material was secured and prepared according to the protocol described in the previous article [16]. Isolation of total RNA from tissue and exosomes was performed with the same type of isolation kits as before. Qualitative and quantitative assessment of the RNA was performed by spectrophotometry (260/280 nm), using an Eppendorf BioPhotometerTM Plus apparatus (Eppendorf, Hamburg, Germany).

### 2.3. Assessment of the Relative Level of Genes and miRNAs Expression (RQ)

The genes were subjected to reverse transcription (RT) using a High-Capacity cDNA Reverse Transcription Kit (Applied Biosystems, Carlsbad, CA, USA) in a Personal Thermocycler (Eppendorf, Germany). The relative expression level of the study genes was analyzed in a 7900HT Fast Real-Time PCR System (Applied Biosystems, Carlsbad, CA, USA) using TaqMan assay for the genes *CCR7* (Hs01013469_m1), *CCL19* (Hs00171149_m1), and *ACTB* (Hs99999903_m1), used as an endogenous control. The reverse transcription (RT) for miRNA was carried out using a TaqMan^®^ MicroRNA Reverse Transcription Kit (Applied Biosystems, Carlsbad, CA, USA) according to the manufacturer’s protocol. The RT reaction was performed in a Personal Thermocycler (Eppendorf, Germany). The components of reactions and conditions were kept as before.

The relative expression level of the study genes was evaluated by the delta-delta CT method (TaqMan Relative Quantification Assay software, Applied Biosystems). RNA from high quality control standard lung tissue (Human Lung Total RNA, Ambion^®^, Austin, TX, USA) was used as a calibrator. 

The relative RQ value of the studied miRNAs was assessed by global normalization. Calibration was performed using commercial RNA (Human Lung Total RNA, Ambion^®^, Austin, TX, USA), and the median CT of those assays (on a per sample basis) was used to evaluate the expression level of the studied miRNAs in tumor tissue and serum. The level of calibrator expression was regarded as RQ = 1.

### 2.4. Statistical Analysis 

The data was analyzed using the Mann–Whitney U-test and/or the Kruskal–Wallis test depending on the size of the groups. The Spearman rank correlation coefficient was used to measure the direction and strength of the association for individual variables. All analyses were performed using Statistica 13.1 software (StatSoft, Cracow, Poland). For all statistical analyses, the level of statistical significance was adopted at *p* < 0.05. RQ values for the study genes/miRNAs were presented as median values.

## 3. Results

### 3.1. Relative Expression Levels of the Studied mRNA in Tumor Tissue (NSCLC) vs. Control Lung Tissue

**Figure 1:**
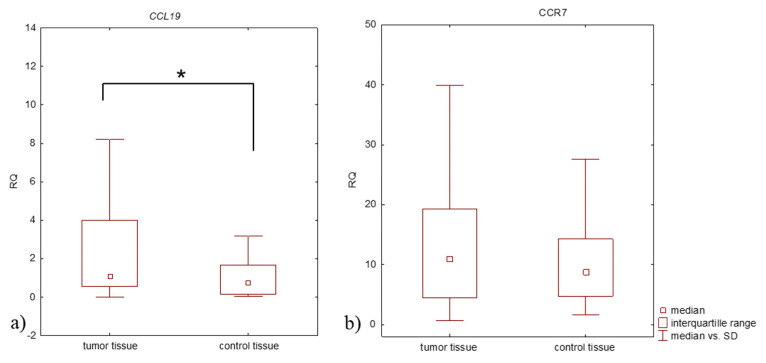
Box plot showing differences in RQ medians for (**a**) *CCL19*, (**b**) *CCR7* in tumor and control tissue in patients with NSCLC (Mann-Whitney U-test); * *p* < 0.05.

*CCR7* mRNA expression was upregulated (RQ > 1) in two study groups: lung cancer tissue (98%) and macroscopically unchanged lung tissue (100%). Also, higher expression levels of *CCR7* mRNA were observed in tumor tissue compared to the control tissue (median RQ: 11.268 and 8.747, respectively), but this difference was not statistically significant (*p* > 0.05, Mann-Whitney U-test) (Figure 1). 

*CCL19* mRNA expression level was upregulated (RQ > 1) in 53% of lung cancer tissue samples and in 40% of macroscopically unchanged controls. A statistically significant difference was observed in the *CCL19* mRNA expression level between tumor tissue (NSCLC) and control tissue (*p* = 0.019, Mann-Whitney U-test), with a higher *CCL19* mRNA expression level in tumor tissue (median RQ: 1.052 and 0.709, respectively) (Figure 1).

### 3.2. Relative Expression Levels of CCR7 and CCL19 mRNA in Tumor Tissue (NSCLC) According to Biological Features and Smoking History of Study Patients

The expression level of *CCR7* mRNA in tumor tissue was higher in female patients compared to the male group (median RQ: 13.548 and 10.796, respectively), as well as in patients aged ≤65 compared to those aged >65 (median RQ: 12.954 and 10.634, respectively), but no statistically significant differences were observed (*p* > 0.05, Mann-Whitney U-test) (Table 2).

The expression level of *CCL19* mRNA was higher in male patients compared to female ones (median RQ: 1.076 and 0.853, respectively) but no significant differences were noted (*p* > 0.05, Mann-Whitney U-test). No statistically significant differences in *CCL19* mRNA expression level were observed according to age. Slightly higher *CCL19* mRNA expression level was found in those aged ≤65 than >65 (median RQ: 1.086 and 1.052, respectively).

Regarding smoking status, higher expression levels of the mRNA were observed in patients who smoked ≥40 PYs than those who smoked <40 PYs; however, this difference was not statistically significant (*p* > 0.05, Kruskal-Willis test) (Table 2). The greatest *CCR7* and *CCL19* mRNA expression level was observed between patients who smoked ≥40 PYs compared to non-smokers (Table 2). 

### 3.3. Relative Expression Levels of CCR7 and CCL19 mRNA in Tumor Tissue (NSCLC) According to Histopathological Assessment and the TNM/AJCC Staging System

Regarding histopathological type, higher *CCR7* and *CCL19* mRNA expression levels were observed in the AC group compared to the SCC group (Table 2); however, no statistically significant differences were observed (*p* > 0.05, Mann-Whitney U-test). 

Similarly, while the greatest *CCR7* and *CCL19* mRNA expression levels were observed in stage III, and the lowest in stage II (Table 2), no significant correlations with cancer stage were observed according to AJCC (*p* > 0.05, Kruskal-Willis test). 

Lower *CCR7* and *CCL19* mRNA expression levels were observed among patients with metastases to lymph nodes (N1 + N2), compared to those without them (N0). However, these differences were not statistically significant (*p* > 0.05, Mann-Whitney U-test) (Table 2).

No significant correlations were observed between *CCR7* and *CCL19* mRNA expression levels and tumor sizes according to pTNM staging (*p* > 0.05, Kuskal-Wallis test). The highest *CCR7* and *CCL19* mRNA expressions were observed in a patient with T1 and the lowest in those with T2 (Table 2).

### 3.4. Relative Expression Levels of the Study miRNAs in Tumor Tissue vs. Serum Patients with NSCLC 

**Figure 2:**
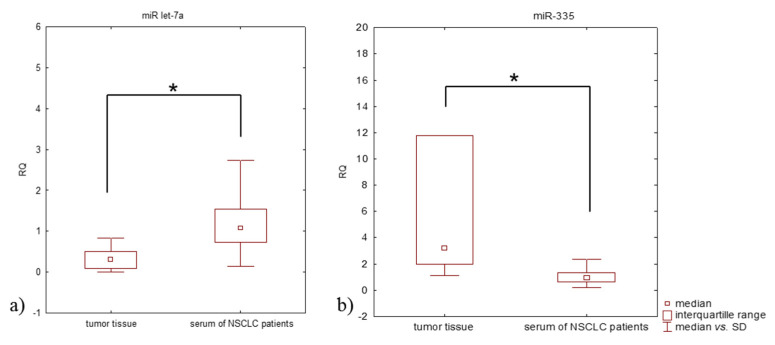
Box plot showing differences in median RQ values for (**a**) miR let-7a, (**b**) miR-335 in tumor tissue and in serum of NSCLC patients; * *p* < 0.05.

All (100%) tumor tissues demonstrated downregulated miR let-7a expression (RQ < 1) and upregulated miR-335 expression (RQ > 1). In addition, 43% of the serum samples from patients with NSCLC demonstrated a downregulated miR-let-7a expression level and upregulated miR-335. As well, the relative expression level of miRNAs in tumor tissue was found to be significantly different to those in the serum of NSCLC patients (*p* = 0.0001, Mann-Whitney U-test) (Figure 2). A lower miR let-7a expression level was observed in tumor tissue compared to serum (median RQ: 0.309 and 1.077, respectively); however, miR-335 expression level was higher in tumor tissue than in serum (median RQ: 3.234 and 0.929, respectively).

### 3.5. Relative Expression Levels of miR-335 and miR let-7a in Tumor Tissue (NSCLC) According to Biological Features and Smoking History 

Regarding sex, a lower expression level of miR let-7a and higher expression level of miR-335 were observed in the group of men compared to women (Table 2); however, no significant differences were found between the study groups (*p* > 0.05, Mann-Whitney U-test).

Patients aged >65 demonstrated a lower miR let-7a expression level and higher miR-335 expression level compared to those aged 65 years and under (Table 2); however, the differences were not statistically significant (*p* > 0.05, Mann-Whitney U test).

Regarding smoking status, higher miR let-7a expression level was noted in patients consuming ≥40 PYs compared to those consuming <40 PYs (median RQ: 0.344 and 0.305, respectively), with the lowest being observed in never-smokers (median RQ—0.198); in addition. In contrast, the highest miR-335 expression level was found in never-smokers, and the lowest in patients consuming ≥40 PYs (Table 2). No statistically significant differences in the expression level of any studied miRNAs were observed with regards to smoking history (*p* > 0.05, Kruskal-Willis test)

### 3.6. Relative Expression Levels miR-335 and miR let-7a in Tumor Tissue (NSCLC) Classified According to Histopathological Classification and the TNM/AJCC Staging System 

No statistically significant differences in miRNA expression levels were observed with regard to histopathological classification (*p* > 0.05, Mann-Whitney U-test). In SCC, the miRNA-335 expression level was slightly elevated, but miR let-7a expression level was decreased (Table 2). 

Patients with stage I (AJCC) demonstrated the lowest miR let-7a expression level (median RQ values) and the highest miR-335 expression level (median RQ: 0.250 and 4.009, respectively). The highest miR let-7a expression level and lowest miR-335 expression level were observed in stage II (median RQ: 0.375 and 2.668, respectively). The highest miR let-7a expression was observed in stage II, followed by stage III, and then stage I. Conversely, the highest miR-335 expression was noted in stage I, followed by stage III, and then stage II. No statistically significant differences in miR-335 and miR let-7a expression levels were observed according to AJCC stage (*p* > 0.05, Kruskal-Willis test) (Table 2).

No statistically significant differences in miR-335 expression level were observed in the tissues of patients with NSCLC with regard to lymph node involvement-N (N0, N1 + N2) (*p* > 0.05, Mann-Whitney U-test). Lower miR-335 expression level and higher miR let-7a expression level were observed in patients with lymph node metastases (N1 + N2), compared to non-metastatic patients (N0) (Table 2).

No significant correlations were found between miR-335 expression level and tumor size (T) according to pTNM staging (*p* > 0.05, Kuskal-Wallis test). The highest miR-335 expression level was observed in a patient with T1 and the lowest with T2; this was opposite to the trend observed for miR let-7a (Table 2).

### 3.7. miRNA Expression Levels in Serum from Patients with NSCLC vs. Control 

**Figure 3:**
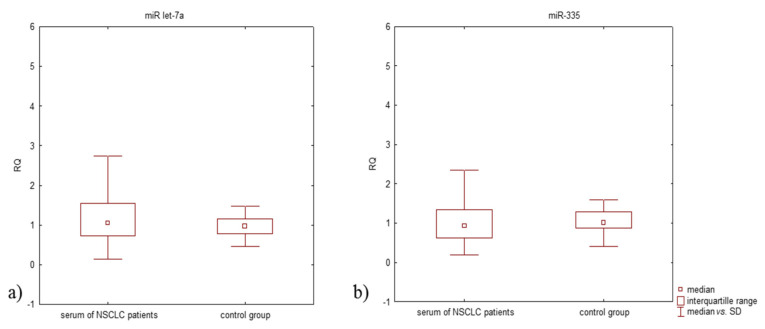
Box plot showing differences in median RQ values for (**a**) miR let-7a, (**b**) miR-335 in the serum of NSCLC patients and in the control group (U-Mann-Whitney test).

The NSCLC patients demonstrated higher miR let-7a expression level compared to controls (median RQ: 1.077 and 0.980, respectively) but lower miR-335 expression level (median RQ: 0.929 and 1.021, respectively) (Figure 3). No statistically significant differences in miR-335 and miR let-7a expression levels were observed in the study groups (*p* > 0.05, Mann-Whitney U-test).

### 3.8. miR-335 and miR let-7a Expression Levels in Serum of Patients with NSCLC According to Biological Features and Smoking History 

Higher miR-335 expression levels were observed among men than women (median RQ: 0.950 and 0.774, respectively), while higher miR let-7a expression level was found among women compared to men (median RQ: 1.292 and 1.053, respectively). No significant differences in miRNA expression levels were found between the two groups (*p* > 0.05, Mann-Whitney U-test). 

The patients aged ≤65 demonstrated lower miR let-7a expression levels but higher miR-335 expression levels than in those aged >65 (Table 3). No statistically significant differences were observed between the two groups (*p* > 0.05, Mann-Whitney U-test). 

Regarding smoking status, higher miR let-7a expression was found among patients consuming <40 PYs than in those consuming ≥40 PYs (median RQ: 1.303 and 0.937, respectively). In addition, higher miR-335 expression level was found in patients consuming ≥40 PYs than in those consuming <40 PYs (median RQ: 1.067 and 0.768, respectively). No statistically significant differences were observed between the two groups (*p* > 0.05, Mann-Whitney U-test). 

### 3.9. miR-335 and miR let-7a Expression Levels in the Serum of Patients with NSCLC According to Histopathological Classification and the TNM/AJCC Staging System 

Significant differences in miR-335 or miR let-7a expression levels were observed according to NSCLC histopathological type (*p* = 0.443 and *p* = 0.040, respectively, Mann-Whitney U-test). Higher miRNA-335 expression was observed in SCC compared to AC (median RQ: 0.993 and 0.774, respectively). Conversely, lower miR let-7a expression level was decreased in SCC compared to AC (median RQ: 1.007 and 1.292, respectively). 

Regarding the AJCC staging system, the lowest miR-335 expression level was observed in a patient with stage I (Table 3) and the highest in stage III. Conversely, the highest miR let-7a expression level was observed in stage I and the lowest in stage III. No statistically significant differences in the expression level of any studied miRNA were observed with regards to AJCC stage (*p* > 0.05, Kruskal-Willis test). 

In the serum from patients with NSCLC, miR-335 expression level was found to vary significantly depending on metastasis to nearby lymph nodes (N) (*p* = 0.021, Mann-Whitney U-test). A higher miR-335 expression level was observed in patients with lymph node metastases (N1 + N2) than in non-metastatic patients (N0) (median RQ: 1.230 and 0.813, respectively). A significantly higher miR let-7a expression level was observed in N0 vs. N1 + N2 (median RQ: 1.230 and 0.813, respectively, *p* = 0.026, Mann-Whitney U-test) (Table 3).

No significant correlations were found between miR-335 expression level and primary tumor size (T) according to pTNM staging (*p* > 0.05, Kuskal-Wallis test). The highest miR-335 expression was observed in a patient with T3 and the lowest in T1 (median RQ: 0.972 and 0.903 respectively). Conversely, the highest miR let-7a expression level was observed in a patient with T1 and the lowest in T3 (median RQ: 1.107 and 1.030, respectively) (Table 3).

### 3.10. Correlation between the Expression Levels of the Studied Genes and miRNAs in NSCLC Patients

A significant negative correlation was found between the expression level of *CCR7* and *CCL19* and miR let-7a in tumor tissue (*p* = 0.0007, rho = −0.426 and *p* = 0.00002, rho = −0.521, respectively, Spearman’s rank correlation). In addition, a significant positive correlation was observed between the expression level of *CCR7* and miR-335 (*p* = 0.0006, rho = 0.430, Spearman’s rank correlation) and between the expression level of *CCL19* and the currently studied miRNA (*p* = 0.00002, rho = 0.524, Spearman’s rank correlation). 

## 4. Discussion

Despite major advances in lung cancer treatment, no progress has been made in its early diagnosis or prediction of progression. Currently, nodal status is considered as one of the most powerful prognostic markers for resected NSCLC; therefore, improved detection techniques predicting lymph node metastasis may enable effective treatment planning for lung cancer.

Tumor cell migration shares many similarities with leukocyte trafficking, which is mainly regulated by chemokines and their receptors [2]. The CCL19/CCR7 axis plays a key role in the activation of native T cells, B cells, and dendritic cells (DCs), and the migration of these cells within lymphoid organs [19]. In NSCLC, CCR7 is expressed on the membranes of lung tumor cells, and *CCR7* mRNA expression level correlates with lymph node metastasis [2,5]. Furthermore it was revealed that CCR7 is involved in the regulation of cell apoptosis [5], proliferation, and EMT in lung cancer cells [3].

Our study on the CCL19/CCR7 axis showed higher levels of *CCR7* mRNA expression in NSCLC samples compared to macroscopically unchanged lung tissue (control tissue), surrounding the tumor. Moreover, *CCR7* upregulation was observed in all study samples (tumor cells and control tissue). Our observation was consistent with previous findings [16]. Thus, in addition to the histological changes, excluding macroscopic pathological changes in the tissue surrounding the tumor, molecular and immunological changes doubtlessly also occur with tumor development. This surrounding tissue, macroscopically unchanged, being part of the *molecular surgical margin* (MSM), also participates in the carcinogenic process due to its heterogeneous composition and presence of cancer cells. This area has been found to demonstrate an increased rate of tumor recurrence [20]. Therefore, the surrounding tissue seems to be important in the diagnostic evaluation of NSCLC patients [21,22]. Our result (observed upregulation of the CCL19/CCR7 axis on mRNA expression level), in a macroscopically unchanged area surrounding the tumor, confirms the presence of immunological/molecular variations in this tissue. 

Numerous studies highlight the value of the expression level of *CCR7* mRNA and its ligand as a diagnostic molecular biomarker; however, the prognostic value has not been clearly established [10,23]. In the present study, the highest expression level of *CCR7* mRNA was noted in patients with AJCC stage III; however, no significant differences in mRNA expression levels were found between AJCC stages I-III. Our results were similar to those of Takanaki et al. [2], who reported significant differences in *CCR7* mRNA expression according to disease stage: no expression was observed for stage IA/IB (AJCC staging), and positive expression for stage III. In contrast, Liu et al. [22] reported a negative correlation between *CCR7* mRNA expression levels and AJCC staging, and Itakura et al. [10] indicated that a high *CCR7* mRNA expression level indicates better prognosis for AC patients and may serve as a good prognostic factor. 

CCR7 is recognized as a key factor in promoting metastasis via the lymphatic system. Surpassingly, lower *CCR7* mRNA expression was observed in patients with metastasis (N1 +N2) than in those without (N0). It should be considered whether the elevated *CCR7* mRNA expression observed in AJCC stage I NSCLC patients in our study may indicate the presence of micro-metastases in the lymph nodes. However, the current staging criteria do not take into account the presence of micro-metastases in lymph nodes identified during assessments of lung cancer [23]. The increased expression of *CCR7* mRNA observed in AJCC stage I NSCLC patients can also be understood as a “hazard signal” resulting in the activation of inflammatory mediators, leukocyte migration, or tissue damage. This hypothesis has been confirmed by other authors [24], who propose that the chemokine system is used as an antitumor immune response. 

CCR7 and its ligand CCL19 control the targeting of T cells and dendritic cells to areas of the lymph nodes where the adaptive immune response is initiated. Recent studies have also shown that CCL19 has potent antitumor properties and inhibits angiogenesis [25]. In the present study, significantly higher *CCL19* mRNA expression levels were observed in the tumor tissue compared to macroscopically unchanged lung tissue, which is consistent with Liu et al. [22]. In addition, the highest *CCL19* mRNA expression level was noted in a case of a patient with a pTNM stage T1 primary tumor, without metastasis to lymph node (N0). It is possible that in the case of non-advanced tumors, increased *CCL19* mRNA expression level may be caused by the infiltration of immune cells at the tumor site during the first stage of antitumor immunological reaction. 

Itakura et al. [10] reported that high CCL19 expression level may also be a good prognostic factor, especially for AC patients. However, Liu et al. [22] note that elevated *CCL19* mRNA expression level positively correlated with the presence of histologically confirmed lymph node metastasis and AJCC staging. In our present study, the highest *CCL19* mRNA expression level was observed in stage III, followed by stage I, and then stage II. We therefore propose that the highest *CCL19* mRNA expression may serve as a poor prognostic factor; however, this observation should be confirmed in larger groups of patients.

Smoking is considered one of the major risk factors of NSCLC development. However, little is known of the relationship between *CCR7* and *CCL19* mRNA expression and smoking history in NSCLC patients. Our study revealed higher *CCR7* and *CCL19* expression levels in patients who smoked ≥40 PYs than those who smoked <40 PYs. It was found that smoking may correlate with elevated *CCR7* mRNA expression level [26], that CCR7 plays a major role in modulating inflammatory responses in airways in pulmonary diseases [27], and that cigarette smoking upregulates *CCR7, CCL19*, and *CCL21* mRNA expression levels in lymph nodes of wild-type mice [28]. It was also concluded that exposure to cigarette smoke can upregulate the number of DCs and cause immunoreactions through CCR7-mediated chemotaxis [29]. Surpassingly, in the NSCLC patients in the present study, higher *CCR7* and *CCL19* expression levels were observed in non-smokers than in smokers; indeed, cigarette smoke extract (CSE) was found to suppress CCR7 expression on DCs in the lungs of smokers, but not diminish their migration towards a CCR7 ligand [28]. Our observations regarding *CCR7* and *CCL19* mRNA expression levels in NSCLC smokers are consistent with those of a previous study which found decreased expression of many inflammatory mediators, including several chemokines, in the BAL cells of smokers [30]. This suggests that patients with different smoking histories exhibit a different immune content and tumor immune microenvironment. Reduced *CCR7* mRNA expression is an important mechanism for dendritic cell retention in the inflammatory process in the lungs of smokers during carcinogenesis. 

Regarding the histopathological type of cancer, a higher *CCR7* and *CCL19* mRNA expression level was observed in the AC group compared to the SCC group. These results may indicate that AC has stronger biological aggressiveness. This is consistent with Kawase et al. [31], who reported that AC appeared to have a more aggressive nature, particularly in patients with pT3 or pT4 primary tumor size. The authors noted that patients with an AC histotype were significantly more likely to demonstrate disease recurrence.

miRNAs play a key role in the regulation of many processes underlying carcinogenesis, such as cancer cell development, differentiation, and migration [32,33,34,35]. They perform a regulatory function for numerous genes involved in metastasis, including chemokines and/or their receptors [10,11]. Moreover, miRNAs are believed to be deregulated in human cancers, emphasizing that they play an important role in tumor onset and development. The present study evaluates the expression level of miR-335 and miR let 7-a, which are known to be regulators of *CCR7* and *CCL19* mRNA expression [36,37]. 

MiR let-7a was identified as an *oncosuppressor miR*, i.e., a tumor-suppressing miRNA [38], and miRNA-7a is known to demonstrate a different expression profile based on the tumor type. Jeong et al. [39] reported lower miR let-7a expression levels in tumor NSCLC tissue compared to control tissue. Our present findings indicate miR let-7a downregulation (RQ < 1) in all NSCLC tissue samples, and that miR let-7a expression level was downregulated (RQ < 1) in 43% of serum NSCLC patient samples. Interestingly, miRNA-7a expression was significantly reduced in tissue compared to serum. Our observation is consistent with those of Jeong et al. [39] regarding NSCLC patients. This suggests that miR let-7a plays a significant role in the local tumor specific regulation of targeted genes involved in lung carcinogenesis. It should be pointed that miR let-7a affects many genes which play a key role in cell pathophysiology, including carcinogenesis. Downregulation of miR let-7a in lung cancer cells is associated with greater self-sufficiency in growth signals, insensitivity to antigrowth signals, limitless replicative potential, and the ability to avoid immune surveillance [34]. However, the full mechanism of miR let-7a regulation in NSCLC remains unclear. Research suggests that miR let-7a may regulate proliferation, apoptosis, and invasion in lung cell lines [38,40,41]. 

Of course, the statistically significant differences in the miR let-7a expression level between the tissue samples and serum exosomes observed in our study should not be surprising. Indeed, miRNA expression is tissue-specific, as confirmed in previous studies based on unrelated human tissues and their exosomal origin [42,43,44]. 

Our findings indicate a negative correlation between the expression level of miR let-7a obtained from serum exosomes and the stage of cancer development according to the AJCC and pTNM classifications. Significantly lower expression levels of the studied miRNAs were observed in the serum of a patient with lymph node metastasis, and this correlation was not observed in tumor tissue. The expression level of miR let-7a in the serum of NSCLC patients was previously found to correlate with disease progression, i.e., the stage of cancer and metastasis to lymph nodes [16]. Our data are consistent with that obtained in a meta-analysis performed by Pop-Bica et al. [45], who found miR let-7a to be downregulated in different sample types (tissue, FFPE tissue, serum, serum/plasma, or exosomes) and to predict a poor outcome in NSCLC patients. This data, and those of our present findings, indicate that miR let-7a expression level may serve as a prognostic biomarker in the liquid biopsy of these NSCLC patients.

To identify non-invasive diagnostic markers, the present study compared the expression of miR let-7a in sera from NSCLC patients and healthy controls. No statistically significant differences were found in the miR let-7a expression level between the study groups; however, higher expression was observed in the NSCLC patients. In contrast, Ying et al. [46] reported significantly lower expression levels of miR let-7a in serum from NSCLC patients compared with healthy controls, and noted that the panel for miRNAs including miR let-7a is able to detect NSCLC development in the early stage of disease. Some differences exist between our present findings and those of other studies; however, these may be due to a range of factors including patient infection, peripheral inflammation, smoking status, epigenetic modification of miR let-7a, or differences in patient numbers.

miRNA expression has been found to play a significant role in smoking-mediated oncogenic events in cancer and their diagnostic/prognostic potential [34,47,48]. It is well-known that smoking is considered a carcinogenesis risk factor, especially in the lung [49]. Therefore, our present study examines the relationship between the miR let-7a expression level in tumor tissue and the number of cigarettes smoked (<40 PYs vs. >40 PYs). It is commonly known that most miRNAs demonstrate varying expression through all carcinogenetic stages, from the normal bronchi of non-smokers to invasive SCC in smokers. Most miRNAs were initially downregulated, in agreement with the hypothesis that miRNA downregulation often occurs in cancer where tissue is losing its normal differentiation. A positive correlation was observed in the present study between the miR let-7a expression level in tumor tissue samples and smoking status. The highest expression level was observed among patients who smoked more than 40 PYs. However, a lower expression level of miR let-7a was observed in the serum of patients consuming ≥40 PYs than those consuming <40 PYs. These results are consistent with those obtained by Rizk et al. [49], who observed a significant negative correlation between miR let-7a expression level and pack years in the serum of smokers. 

The present study also focuses on miR-335 expression level in NSCLC patents, which is involved in the regulation of epithelial-mesenchymal transition (EMT), proliferation, and migration of lung cancer cells [50]. Huo et al. [51] indicated than miR-335 still plays a suppressive role in NSCLC tumorigenesis, and that the downregulation of miR-335 in lung cancer cells promoted cell proliferation through upregulation of Tra2β [52]. Significantly lower miR-335 expression levels were also found in NSCLC tissue samples compared to control lung tissue. In addition, in the present study, the miR-335 expression level was upregulated in all tumor tissues and in 43% of the serum of NSCLC patients. These findings have been confirmed by Scalora et al. [53], who noted an increased expression level of miR-335 in lung cancer cells and confirmed its association with a higher proliferation rate and progression. Unfortunately, our studies did not identify any association between miR355 expression dysregulation and cancer progression. Our analysis did not reveal any correlation between the miR-335 expression level in tumor tissue and stage of cancer development according to AJCC and pTNM classifications. In contrast, Huo et al. [51] found that miR-335 expression level in tumor tissue significantly correlated with lymph node metastasis and pathological TNM stage. 

Moreover, our findings do not confirm any relationship between the miR-335 expression level in tumor tissue samples/serum and the age or sex of study patients. These observations are consistent with those of other researchers [51,54]. 

Interestingly, a negative correlation was identified between the miR-335 expression level in tumor samples and smoking history: a lower miR-335 expression level was observed in smokers compared to non-smokers, and decreased as smoking intensity increased (PYs < 40 vs. PYs > 40). Ong et al. [55] reported that current smokers demonstrated a lower miR-335 expression level in primary lung fibroblasts compared to ex-smokers. Downregulated miR-335 expression may enhance fibroblast proliferation by targeting Rb1, CARF, and SGK3.

Lower miR-335 expression levels were observed in the serum of NSCLC patients compared to controls; however, the differences were not statistically significant. This is the first such report of miR-355 deregulation; however, decreased miR-335 expression level has been noted in the serum of patients with various malignant tumors, such as hepatocellular carcinoma (HCC) [56], gastric cancer [57], and gallbladder cancer (GC) [58]. Ciu et al. [56] reported that HCC patients with lower miR-335 expression levels in serum had significantly poorer prognosis than those with higher serum expression. This observation suggests that miR-335 may have prognostic value. Moreover, Wu et al. [58] noted a negative correlation between the miR-335 expression level and lymph node metastasis in GC patients. Our present findings indicate high miR-335 expression level in serum from NSCLC patients, and that this level correlated with cancer progression based on clinical tumor stage (AJCC), lymph node involvement and tumor size. Hence, miR-335 expression level may serve as a biomarker with negative prognostic value in the liquid biopsy of NSCLC patients. Similarly, Sun et al. [59] indicated that exosomal miRNA-335 enhances invasion and metastasis in colorectal cancer by facilitating EMT via targeting RASA1. 

Our present findings indicate that *CCR7/CCL19* mRNA expression level has a significant negative correlation with miR let-7a expression level and a positive correlation with miR-335. Similar findings regarding the direct regulatory influence of *CCR7/CCL19* mRNA and miR let-7a have been obtained in experiments with Luciferase reporter assays [60]. It has also been documented that miR let-7a directly regulates CCR7 protein expression through interaction with 3′UTR of *CCR7*. 

The limitation of our research was that only small groups of patients were included in the study. This may also be the reason why relatively few statistically significant results were obtained regarding the relationship between the expression level of CCR7/CCL19 mRNA, study miRNAs, and cancer features. Moreover, according to the recommended NSCLC treatment algorithm and indication for surgical treatment (depending on the advancement of the lesion), it was difficult for us to obtain an equal number of tissue samples for study groups (e.g., I-III according to TNM/AJCC staging).

## 5. Conclusions

Changes in the level of CCL19/CCR7 mRNA expression in tumor tissue can be considered as a diagnostic marker for NSCLC patients. The CCR7 and its ligand CCL19, play two important but challenging roles in NSCLC. On the one hand, the CCR7/CCL19 axis has a key role in promoting metastasis via the lymphatic system. On the other hand, the CCL19/CCR7 axis is involved in the modulation of the immune response in a growing tumor. It should be noted that the role of CCL19/CCR7 in NSCLC appears to be complex, and post-transcriptional regulation of these genes may be important in the progression and metastasis of this cancer. 

Significant differences in miR let-7a and miR-335 expression levels were found between the tumor tissue and serum of NSCLC patients, indicating that miR let-7a has a suppressive function in the primary lesion and that miR-335 has an oncogenic one. Our findings confirm previous observations indicating miR-335 and miR let-7a expression levels in the serum of patients with NSCLC correlate with lymph node metastasis. The studied miRNAs may potentially serve as non-invasive molecular biomarkers with prognostic value (prediction of metastatic potential) in liquid biopsy. 

## Figures and Tables

**Table 1:** Demographic characteristics of patients and features of lung cancer.

Patient Characteristics	Number of Patients (%)
Sex	
Female	25 (41.67%)
Male	35 (58.33%)
Age	
≤65	28 (46.67%)
>65	32 (53.33%)
Smoking status and smoking history	
<40 PYs ^a^	26 (43.33%)
≥40 PYs	26 (43.33%)
Non-smoking	7 (11.67%)
**Tumor Characteristics**	**Number of Cases (%)**
Histopathological type of NSCLC	
SSC	28 (46.67%)
AC	32 (53.33%)
AJCC ^b^ staging system	
Stage I	30 (50%)
Stage II	21 (35%)
Stage III	9 (15%)
Metastasis to lymph nodes spread according to the pTNM ^c^ staging system	
N0	39 (65%)
N1 + N2	21 (35%)
Tumor size according to the pTNM staging system	
T1a + T1b	21 (35%)
T2a + T2b	29 (48.33%)
T3	10 (16.67%)

^a^ PYs—pack years, 1 Pack Year = 20 cigarettes smoked per day for one year; according to the NCI Dictionary of Cancer Terms [17]; ^b^ AJCC—American Joint Committee on Cancer Staging according to the IASCLC Staging Project 7th ed. (2010) Cancer; ^c^ pTNM—post-operative Tumor Node Metastasis staging system according to the WHO Histological Typing of Lung Tumor.

**Table 2:** Clinical and pathological features: median expression level (RQ value) of evaluated genes and miRNAs in tumor tissue (NSCLC).

Clinical and Pathological Features	*CCR7* (Median RQ)	*p* Value	*CCL19* (Median RQ)	*p* Value	miR let-7a (Median RQ)	*p* Value	miR-335 (Median RQ)	*p* Value
Female	13.548	>0.05	0.853	>0.05	0.328	>0.05	3.053	>0.05
Male	10.796		1.076		0.295		3.554	
≤65	12.954	>0.05	1.086	>0.05	0.410	>0.05	2.455	>0.05
>65	10.634		1.052		0.299		3.423	
<40 PYs	8.510	>0.05	0.712	>0.05	0.305	>0.05	3.366	>0.05
≥40 PYs	11.299		1.210		0.344		2.906	
Non-smoking	13.548		7.425		0.198		5.059	
SCC	9.351	>0.05	1.028	>0.05	0.304	>0.05	3.292	>0.05
AC	13.001		1.227		0.315		3.108	
AJCC		>0.05		>0.05		>0.05		>0.05
Stage I	11.040		1.610		0.250		4.009	
Stage II	10.504		0.601		0.375		2.668	
Stage III	11.315		1.663		0.260		3.848	
pTNM (N)		>0.05		>0.05		>0.05		>0.05
N0	12.907		1.227		0.301		3.319	
N1 + N2	10.504		1.028		0.352		2.844	
pTNM (T)		>0.05		>0.05		>0.05		>0.05
T1	17.897		2.0410		0.198		5.059	
T2	9.351		0.677		0.352		2.844	
T3	12.246		0.899		0.333		3.124	

**Table 3:** Clinical and pathological features: median expression level (RQ value) of evaluated miRNAs in serum (NSCLC).

Clinical and Pathological Features	miR let-7a (Median RQ)	*p*-Value	miR-335 (Median RQ)	*p*-Value
Female	1.292	>0.05	0.774	>0.05
Male	1.053		0.950	
≤65	0.910	>0.05	1.090	>0.05
>65	1.249		0.800	
<40 PYs	1.303	>0.05	0.768	>0.05
≥40 PYs	0.937		1.067	
Non-smoking	1.292		0.774	
SCC	1.007	0.040	0.993	0.044
AC	1.292		0.774	
AJCC		>0.05		>0.05
Stage I	1.169		0.858	
Stage II	1.085		0.922	
Stage III	0.969		1.032	
pTNM (N)		0.026		0.021
N0	1.230		0.813	
N1 + N2	0.813		1.230	
pTNM (T)		>0.05		>0.05
T1	1.107		0.903	
T2	1.085		0.922	
T3	1.030		0.972	
